# Social intuition: behavioral and neurobiological considerations

**DOI:** 10.3389/fpsyg.2024.1336363

**Published:** 2024-04-23

**Authors:** Tjeerd Jellema, Sylwia T. Macinska, Richard J. O’Connor, Tereza Skodova

**Affiliations:** School of Psychology and Social Work, University of Hull, Hull, United Kingdom

**Keywords:** social intuition, implicit learning, mirror neuron mechanism, affective valences, bodily articulations, autism spectrum conditions, autistic traits, anticipation

## Abstract

Social intuition is instrumental in bringing about successful human interactions, yet its behavioral and neural underpinnings are still poorly understood. We focus in this article on the automatic, involuntary, nature of social intuition, rather than on higher-level cognitive and explicit Theory-of-Mind processes (which contribute to rendering social intuition meaningful in real-life situations). We argue that social-affective implicit learning plays a crucial role in establishing automatic social intuition. These implicit learning processes involve associations between the perception of other’s bodily articulations, concurrent events, and the consequences or outcomes in terms of subsequent actions, affective valences and visceral states. The traditional non-social implicit learning paradigms do not allow one to draw conclusions about the role of implicit learning processes in social intuition, as they lack these vital characteristics typically associated with human actions. We introduce a new implicit learning paradigm, which aims to fill these gaps. It targets agile, rapid, social-affective learning processes, involving cue contingencies with a relatively simple structure, unlike the very complex structures that underpin the traditional tasks. The paradigm features matching social and non-social versions, allowing direct comparison. Preliminary data suggest equal performance of TD (typically-developed) and ASC (autism spectrum conditions) groups on the non-social version, but impaired implicit learning in ASC on the social version. We hypothesize that this reflects an anomalous use of implicitly learned affective information in ASC when judging other people. We further argue that the mirror neuron mechanism (MNM), which is part of the Action Observation Network, forms an integral part of the neural substrate for social intuition. In particular as there are indications that the MNM supports action anticipation, and that implicitly learned information can trigger MNM activation, which both seem vital to a social intuition ability. The insights that can be derived from comparing the performances of TD and ASC individuals on (non)social implicit learning tasks, and the implications for the role of MNM activation, are discussed.

## 1 Introduction

Successful socio-affective human interactions form the glue that binds society, yet we are only just beginning to unravel the underpinning neural and behavioral mechanisms. Typically-developed (TD) individuals differ greatly in their social interaction abilities, while many psychological and neuro-developmental conditions, and mental illnesses, are characterized by profound difficulties in social interaction. A clear example are individuals with autism spectrum conditions (ASC; Autism Spectrum Conditions, also called Autism Spectrum Disorders; DSM-V-TR, 2022). The juxtaposition of ASC and TD therefore has the potential to offer insight into the mechanisms and processes underpinning socio-affective interactions.

One fundamental requirement for successful interactions is the skill that allows one to make rapid assumptions about what actions others are likely to take and what their intentions, emotions and thoughts might be. These assumptions should be made in a fairly effortless, automatic, manner, without the involvement of any prolonged, deliberate and effortful reasoning. We commonly call this skill *social intuition*. Social intuition manifests itself as ‘immediate insight’, and enables the rapid, social decision-making necessary to successfully navigate the ever-changing socially interactive world (Bechara et al., 1997; Lieberman, 2000). Task demands and time-restraints during social interactions mean that there is simply no time available to consciously reflect on what judgment to make or action to take. Therefore, individuals who depend upon deliberate, effortful reasoning, are likely unable to keep up with the pace of interactions. This may cause them to quickly lag behind and feel inadequate in social interactions. We propose this happens in ASC.

Social intuition can be considered a subdivision of social cognition. Whereas social cognition generally is concerned with how people, both explicitly and implicitly, process and apply information about other people and social situations (Frith, 2008), social intuition is essentially limited to implicit, automatic, effortless, processes that help to steer one’s behavior in social situations in an advantageous way (Lieberman, 2000). The topicality of the question of how we accomplish the complex task of navigating the social world is reflected by recent propositions for a ‘second-person’ or ‘interactive’ approach in social cognition and social neuroscience (Schilbach et al., 2013; Redcay and Schilbach, 2019). This approach typically abandons the traditional use of 2D displays (images and videos), and replaces this with real-world real-time scenarios involving live actors, which brings social intuition to the forefront.

There are three main parts to this article. The first part [2] focusses on the role of implicit learning in forming social intuition. Here we look at the main non-social implicit learning paradigms, and indicate why they are of limited relevance for social interaction [2.1]. These non-social paradigms are then contrasted to social implicit learning paradigms, involving affective valences and bodily articulations [2.2]. We examine whether automatic action anticipation – a crucial ingredient of social intuition – can be brought about by implicit learning [2.3], and compare the performance of individuals with ASC on social versus non-social implicit learning tasks [2.4]. We next look at recent developments that aim to incorporate affective valences and bodily articulations in implicit learning paradigms [2.5].

In the second part [3], we discuss future developments and introduce a new paradigm developed in our lab, where implicit learning is tested in a social version [3.1] and in a matching, non-social, version [3.2]. Preliminary results, interpretations, alternative explanations and relevance are discussed [3.3].

In the third part [4], we examine the neural basis of social intuition. We look in particular at the role the mirror neuron mechanism (MNM) may play in social intuition, and posit that action anticipation is implemented in the MNM, and, importantly, that action anticipation can be triggered by implicitly learned action cues [4.1].

## 2 Implicit learning as a means to acquire social intuition

It has been suggested that social intuition is supported by a toolbox of social learning heuristics (Hertwig et al., 2013), many of which are *implicit* in nature. This means that one is not aware that any learning has taken place, even though it clearly affects one’s subsequent decision making. Implicit learning typically applies to knowledge about rule-governed complexities in the environment and received a lot of attention (Reber, 1989; Bargh, 1994; Cleeremans, 2011; Schilbach et al., 2013). Although implicit processing and implicit knowledge proved difficult to define (see Gómez et al., 2017, for a discussion), one or more of the following adjectives are typically used to describe it: unintentional, uncontrolled, goal-independent, automatic, stimulus-driven, unconscious, efficient, effortless, fast and inflexible (Shiffrin and Schneider, 1977). From this list it is clear that a number of different dimensions are involved. Rather than each dimension forming a necessary constituent, what may matter most for defining implicitness is their relative contribution (Bargh, 1994), with unawareness (Moors and De Houwer, 2006) and automaticity (De Houwer and Moors, 2012) probably the most cited features.

Explicit learning processes, which often involve a form of deliberate hypothesis testing, are described by the opposite adjectives: intentional, controlled, goal-dependent, deliberate, top-down driven, conscious, inefficient, effortful, slow and flexible. Implicit and explicit processes may operate independently (dual process operation, Kliemann et al., 2013), and may be based on different mechanisms. This is supported by findings that the underpinning neuronal circuits may be dissociable, with implicit tasks showing in particular activation in the striatum and basal ganglia (e.g., implicit sequence learning; e.g. Rauch et al., 1997), anterior cingulate cortex and cerebellum, and explicit tasks showing in particular activation of prefrontal and visual cortical areas (Aizenstein et al., 2004). The relevance of the implicit-explicit distinction has been introduced very effectively to the wider public by Kahneman (2011) in his book “Thinking, Fast and Slow”.

### 2.1 Non-social implicit learning paradigms

Humans seem to have the capacity to extract, and use, knowledge they apparently have no conscious access to. It could be that this knowledge was initially acquired explicitly and consciously, but was subsequently forgotten. Yet, the knowledge did not vanish from the brain, and kept on exerting an unconscious influence on behavior and decision making. However, it could also be that the knowledge never reached consciousness in the first place, but was learned implicitly.

With respect to this latter route, the literature shows an abundance of implicit non-social learning studies, demonstrating that one can learn about the sequential structure or rule that governs non-social, artificial, stimuli presentations (such as dots or letters), without one being explicitly aware of this underlying structure or rule. These non-social implicit learning tasks typically require hundreds of trials to induce a learning effect. Often cited examples are the Serial Reaction Time (SRT) task and the Artificial Grammar Learning (AGL) task. In typical SRT tasks (Nissen and Bullemer, 1987) stimuli are presented in one of four quadrants on a screen, and participants need to give a speeded response that corresponds to the specific quadrant where the stimulus appeared. Even though participants show no awareness or knowledge about the sequence, they consistently improve their reaction times over the course of the task, suggesting they had unconsciously learned the underlying pattern. In AGL tasks (Reber, 1967), participants are first exposed to meaningless strings of letters (e.g., XMXTVM; XXTRM) that are structured by a hidden, complex, rule (i.e., an artificial grammar). If, in a subsequent test phase, participants are able to accurately judge whether new strings follow the artificial grammar or not, then that suggests that they implicitly learned the underlying grammar (Reber, 1967; Dienes and Scott, 2005). Crucially, participants who implicitly learned the grammar often claim to respond at random, or to rely on an intuition they cannot further explain (Dienes and Scott, 2005).

The processes of unconscious knowledge acquisition, through implicit/unconscious learning, that emerge from these implicit learning paradigms, have been proposed to affect human behavior not only in laboratory paradigms, but also in the real-world social domain (Hudson et al., 2012b; Jurchiș and Dienes, 2023). However, this poses some serious problems. The non-social, implicit learning paradigms, that measure the capacity to implicitly pick-up on complex regularities between artificial, meaningless stimuli (dots, isolated letters), may be of no value for social interactions nor for social cognition in general. Such stimuli have no social significance, and the very large number of repetitions required to learn the regularities may render it impractical to be relevant for social interactions (though situations are conceivable where exposure to social ‘rules’ results in implicit learning only after large number of repetitions). Last but not least, social interactions are characterized, and guided by, affective valences, and by attitudes and dispositions, associated with human actions. All of these are wholly missing in the non-social paradigms.

### 2.2 Social implicit learning paradigms: affective valences and bodily articulations

Greenwald and Banaji (1995) defined implicitly learned material as introspectively unidentified (or inaccurately *identified*) traces of past experience that mediate favorable or unfavorable feeling, thought, or action toward objects. This definition acknowledges the role of affective valences in implicit learning. It has been argued that for cognition to be fully effective, it is not enough that the agent is able to understand and predict developments in the environment, it must also care about them, it must *desire* certain types of outcomes and *shun* others (Cleeremans, 2011). In this view, emotions are computational tags that subserve and facilitate cognitive processes. This may be true not only for explicit learning but also, and maybe especially, for implicit learning. It has been shown that an affective valence gets attached to a stimulus also when that stimulus is not consciously recognized (Kunst-Wilson and Zajonc, 1980). These implicitly associated affective valences may well be essential to make the rapid, intuitive, decisions that are beneficial to the individual and ultimately support their survival (Cleeremans, 2011). This view, in which emotions are computational tags that subserve and facilitate cognitive processes, is somewhat reminiscent of Damasio’s somatic marker hypothesis (Damasio, 1994).

During real-life social interactions, the relevant stimuli usually consist of the other’s actions, gestures, facial expressions and vocalizations, i.e., bodily articulations of one sort or another. Picking up on regularities and contingencies between the occurrence of these bodily articulations, contextual cues, and the immediate consequences (in terms of subsequent actions and/or subsequent reward/punishment) is an important source for implicit learning about others. Bodily articulations and affective valences thus seem necessary ingredients in any future implicit social learning paradigm.

There are a few cases where an attempt was made to include the social domain in an implicit learning paradigm (e.g., Brown et al., 2010; Travers et al., 2013). However, in these cases, the social nature of the animate objects was not pertinent to the paradigm and could just as well have been replaced by inanimate objects (i.e., affective valences, attitudes and dispositions played no part). One striking example of implicit social learning though was reported by Bayliss and Tipper (2006), who found that participants who had been presented with different actors who either consistently looked at, or away from, the location where a target would appear, subsequently judged those actors that had looked away from the target as the least ‘trustworthy’ and those who had looked at the target as the most ‘trustworthy’. Crucially, in a debrief, participants did not recall any actor-gaze-cue contingencies, suggesting the contingencies had *implicitly* affected their social judgments. A further interesting finding was that this effect correlated negatively with the participants’ scores on the Autism Quotient (AQ; Baron-Cohen et al., 2001).

### 2.3 Anticipation of others’ actions based on implicitly learned cues

A social intuition, revealing itself as a sudden insight, inclination or drive relating to another individual, is typically prompted by the observation of an action or bodily articulation performed by that individual. However, it would be even more advantageous if one would be able to anticipate the other’s action and then for that anticipation to prompt the intuition. That would save precious time, which can be used to prepare one’s response in accordance with the intuition, or to coordinate one’s own actions with those of others (Csibra, 2008). Especially in the fast-paced social interactions, this would be very beneficial. The automatic anticipation of others’ actions is therefore thought to be an essential building block of social intuition (Hudson et al., 2009, 2012a; Krol et al., 2020). But what could be the source of such an anticipation, what could it be based on? First of all, actions typically do not occur in isolation, but in chains, where one sub-action is immediately followed by another sub-action and so on, resulting in an action-chain that serves a purpose (achieves a goal). Reaching out for a cup of coffee, bring it to the mouth, take a gulp and place the cup back. Once the first action of the chain started, it is able to trigger the entire action-chain almost instantaneously, like a row of falling dominos, unfolding ahead of real-time developments, and thus allowing the observer a glimpse of the future. The triggering event could also precede the action, it could for example be formed by the other’s initial attention being directed at the object (Jellema et al., 2000). In general, others’ actions seldom commence unheralded, they usually are foreshadowed by cues, which can be action cues (as above) or originate in the environment. Environmental/contextual cues can be artificial and explicit, such as a pedestrian traffic light jumping to green (the person waiting is expected to start walking), but can also be subtle and implicit.

Especially in social interactions, cues tend to be subtle and implicit (Amso and Davidow, 2012). Here, learning opportunities are often not explicit, as the learned cue-action contingencies may not reach consciousness (Monroy et al., 2019). The observer may remain completely unaware that any knowledge has been acquired, but the cue will nevertheless induce automatic anticipations in the observer regarding others’ upcoming actions (Braukmann et al., 2017; Monroy et al., 2019). For example, when someone slightly moves in their chair in preparation to stand up, or slightly raise their hand when they are about to say something. Or when a nurse immediately sees what the patient wants to do, or a flight attendant immediately gauges that a passenger is going to cause trouble. Their insights are based on the automatic interpretation of a constellation of often simultaneously occurring cues. However, when one would ask the nurse or flight attendant how they reached their judgment, they may not even be able to recall the exact cues, they just ‘saw’ it. Nevertheless, a lot of implicit learning must have taken place before they were able to pick up on the relevant cues and then just ‘saw’ it (a naïve observer would have been oblivious). Without this implicit learning ability, other’s actions would indeed often come unheralded, with the potential to surprise and bewilder any observer. This is, however, exactly how individuals with ASC, who arguably lack social intuition, often describe their experiences of others’ actions. For them, deciphering the intended meaning of such actions is often anything but effortless and automatic.

### 2.4 Implicit learning in autism

ASC is a pervasive neurodevelopmental condition characterized by atypicality in social communication, impaired social development, and stereotypical, repetitive behaviors, often associated with obsessive interests and a lack of empathy (DSM-V-TR, 2022). Symptom severity varies hugely in ASC; three levels are identified based on severity and need of support. Already at Level 1 (previously called high-functioning autism), which is the least severe level with normal IQ distribution and no, or limited, need for support, the difficulties in social and emotional domains are marked. Intriguingly, it has been suggested that the deficits in the social domain that characterize ASC may have their roots in impairments in implicit (spontaneous) processing, with relatively intact explicit (deliberate) processing (Jellema et al., 2009; Senju et al., 2009; Schilbach et al., 2012). These ideas align with the Dual Process Theory of Autism (Evans, 2003), which proposes a dominance of deliberative (Type 2 processing) over intuitive (Type 1) processing. This proposition seems plausible, as in the social domain it is especially the quick and intuitive interpretation of others’ non-verbal behavior that is problematic in ASC. However, studies aimed specifically at finding out whether implicit learning is impaired in ASC concluded that it is intact (e.g., Gordon and Stark, 2007; Barnes et al., 2008; Brown et al., 2010; Nemeth et al., 2010; Foti et al., 2015). Crucially though, all these studies had one important limitation in common: they focused exclusively on implicit learning in the *non-social* domain, using tasks involving probabilistic sequence rules. The social domain, where implicit learning relies on affective valences associated with the stimuli, and abstract concepts such as dispositions and intentions, was not incorporated. It may well be that different mechanisms are at play in the implicit learning of social, as compared to non-social, information.

### 2.5 Recent advances in the study of social implicit learning

Recently, a few studies aimed to specifically address the limited relevance of the non-social learning paradigms for social interactions (Norman and Price, 2012; Zhang et al., 2020; Costea et al., 2023; Jurchis et al., 2023; Jurchiș and Dienes, 2023). For example, Jurchis et al. (2023) took the approach to try and ‘socialize’ the traditional Artificial Grammar Learning (AGL) task. In the traditional AGL task, strings of meaningless letters are presented in the acquisition phase. In Jurchis et al. (2023), these strings of letters were replaced by strings of emotional facial expressions (all faces in one and the same string belonged to the same identity). As in the traditional AGL task, the strings of faces followed a hidden grammar, determining a number of specific sequences in which the faces were presented. In this way, Jurchis and colleagues were able to subject their implicit social learning paradigm to the same rigorous methods and principles that govern non-social implicit learning paradigms. At the start of the acquisition phase, participants were told that they had to remember the stimuli in each string. At the start of the test phase, they were told that the sequences in which these stimuli were presented had actually been specified by a complex set of rules, and that their task was to determine whether new test strings followed these rules or not. The number of repetitions of strings in the acquisition phase was also dramatically reduced compared to the non-social equivalent. In the test phase, where participants indicated whether the new strings followed the rule or not, in 70.6% of the trials they reported that their judgments were based on implicit/unconscious knowledge (the participants indicated that the remaining judgments were based on explicit knowledge). The implicit/unconscious judgments turned out to be correct in 58.2% of trials, which was significantly above-chance. In a further experiment in Jurchis et al. (2023), and also in Jurchiș and Dienes (2023), the same paradigm was used but with stimuli consisting of strings of martial art poses. These poses were ‘social’ in that human beings were presented displaying meaningful bodily articulations, yet (presumably) lacking in emotional valence. Similarly, in 67.5% of the trials, participants attributed their responses to unconscious knowledge, and in 57.5% of these latter trials the correct answer was given, again significantly above-chance.

Thus, implicit learning could be induced using social stimuli with relatively small numbers of repetitions, both when affective valences were associated with these stimuli and when not. However, these paradigms still do not resemble the patterns and types of stimuli that characterize real social situations, nor are emotional, motivational and interactive aspects of real social situations included. A few issues in particular spring to mind when considering the relevance of these tasks for daily-life social intuition. (i) While emotional facial expressions are highly relevant for social interactions, the simultaneous presentation of multiple facial expressions belonging to one and the same individual is impossible to encounter in real-life. Although the task recruits emotional processes, it is not done in an ecologically meaningful way. (ii) The participants were instructed to direct their attention to the very stimuli whose appearance was specified by the hidden rules. In daily-life interactions, the contingencies/regularities between bodily cues and their consequences typically do not form the focus of attention. Rather, attention may be focused on the joint activity (e.g., a conversation) one is engaged in, while the accompanying bodily cues that trigger anticipations may very well not be noted consciously. It is therefore unclear to what extent participants’ behavior in this task informs about what people can learn from regularities/contingencies in real-life situations.

## 3 Future developments in implicit social/affective learning paradigms

In a series of recent experiments, Macinska and Jellema used a new implicit learning paradigm, designed to be particularly relevant for real-world social interaction (Macinska et al., 2015a,b; Macinska and Jellema, 2016; Macinska, 2019). The core paradigm was developed in Hudson et al. (2012b). The implicit social learning induced by this paradigm is characterized by rapid, agile, incidental processes that track relatively low order stimulus combinations, rather than slower processes that track very complex sequences and combinations, as in the traditional implicit learning paradigms. It requires a relatively small number of repetitions (about 10) to induce implicit learning effects, and allows to specifically compare participant’s abilities for *social* versus *non-social* implicit learning. With respect to the latter, a non-social paradigm was designed that matched the social paradigm, as far as possible, in terms of the number of cues, internal structure and difficulty-level. This direct comparison is important as there is still a large gap in our knowledge about the extent to which the distinction between the social and non-social domain is relevant. Thus, in both paradigms a specific *contingency* between three different cues had to be learned. The main difference was, however, that implicit learning in the social paradigm crucially depended on affective valences, which were absent in the non-social paradigm, where learning depended purely on stimulus contingencies. The social cues were deliberately chosen for being straightforward and simple, so that ASC participants should not experience problems in deciphering their meaning. This was done to ensure that possible difficulties in implicit learning of the social contingencies cannot be attributed to difficulties with representing the social information *per se*.

Importantly, in the acquisition phases of both paradigms, contingencies rather than probabilities, have to be learned. That is, a rule determined specific contingencies between coordinated changes in the appearances of three distinct cues (each with two levels). The specific appearance of the level of one cue can be predicted with 100% certainty on the basis of the appearances of the levels of other two cues. It should be noted that in these paradigms, the participant is asked to simply watch the video-clips; there was no attempt to draw their attention to certain stimuli and they were not given a task to do. The acquisition phase was rather short (4 min), so they should be expected to maintain attention throughout that period.

### 3.1 Social version

#### 3.1.1 Acquisition phase

**Figure 1:**
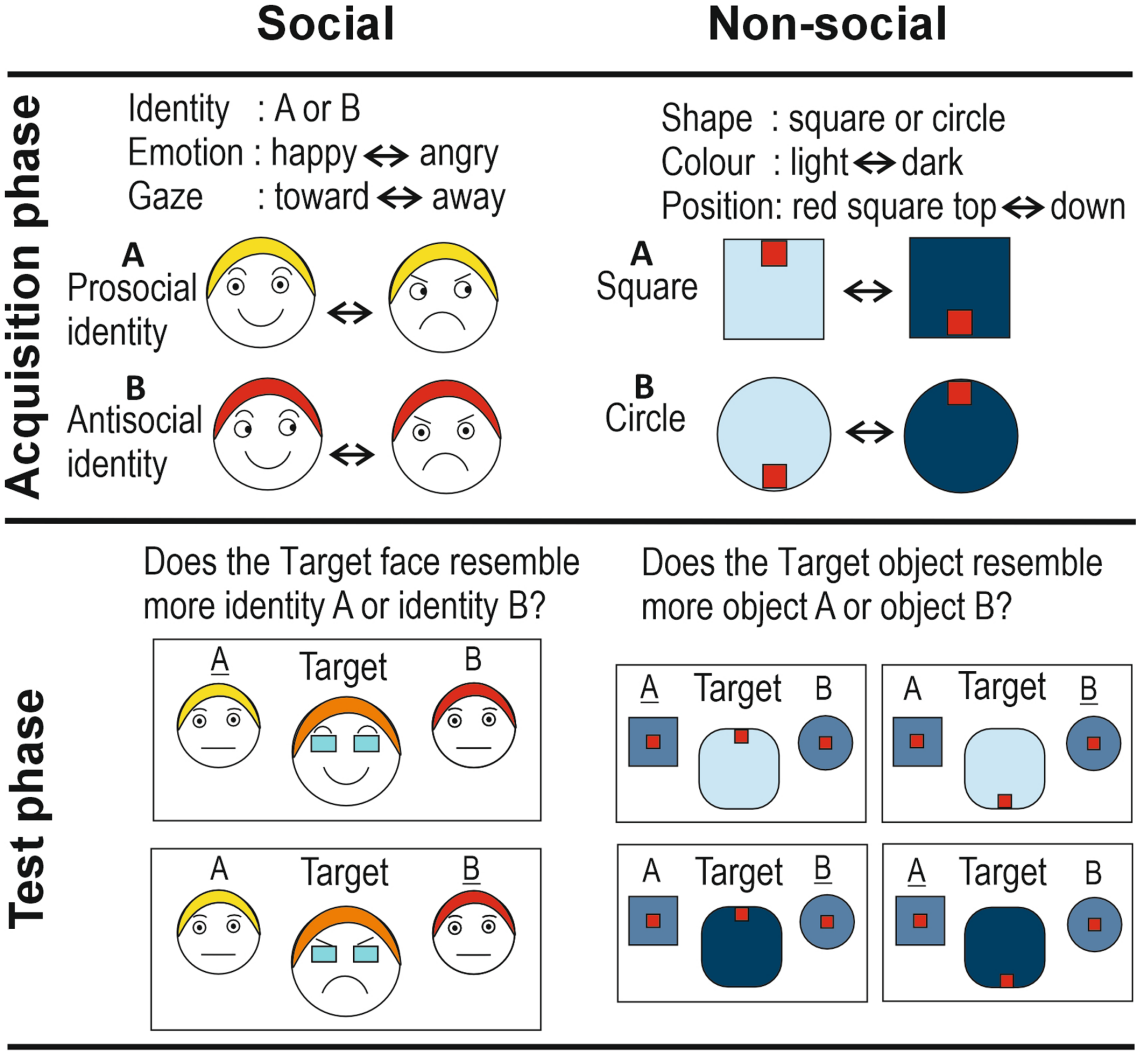
Schematic representation of the social and non-social implicit learning paradigms. The social identities are shown with differently colored hair for the purpose of illustrating their different identities. Responses in the Test phase (‘A’ and ‘B’) that reflect correct implicit learning are underlined.

In the social version, the three cues were: dynamic facial emotional expressions (levels: happiness vs. anger), gradual eye gaze shifts (levels: toward vs. away), and identity (levels: identity A vs. identity B). The advantage of using dynamic facial expressions is that they are more ecologically-valid (Jellema et al., 2011; Palumbo and Jellema, 2013; Palumbo et al., 2015; Sato et al., 2019). Participants were presented with short video clips (2 s) depicting the frontal face view of an actor (agent A or agent B; Figure 1). Their facial expressions and gaze directions changed smoothly over the course of the clips, displaying a natural facial movement. Fifty percent of trials showed actor A, the other 50% actor B, in random order. Actor A started with a happy expression looking straight ahead (at the observer), which then gradually morphed into an angry expression, while simultaneously the eye direction gradually moved away from the observer (so that in the final frame the actor looked angry away from the observer). The clips were also played backwards an equal number of times. Thus, it can be said that agent A had a positive disposition toward the observer. Agent B started with a happy expression looking away from the observer, which gradually morphed into an angry expression, while simultaneously the eye direction gradually moved toward the observer (clips were also played backwards an equal number of times). Actor B thus had a negative disposition toward the observer. Importantly, both actors smiled and frowned for exactly the same amount of time and looked at, and away from, the observer for exactly the same amount of time. This was to ensure that the actor’s pro- or anti-social disposition toward the observer could only be learned on the basis of the specific combination of two cues linked to an identity; the agent’s disposition could not be learned on the basis of each cue on its own. Too many repetitions meant most participants would detect the contingency (i.e., explicit learning), while with too few repetitions no implicit learning might take place.

#### 3.1.2 Test phase

In the subsequent test phase, an indirect measure was used to find out whether any implicit learning had taken place. This measure involved a morph of the facial expressions of the two identities A and B, flanked by the original neutral faces of these two identities. The morphed identity was composed of 60% of the maximally smiling actor A and 40% the maximally smiling agent B (happy morph), and then progressed in steps of 5% toward 40% of the maximally smiling actor A and 60% the maximally smiling agent B. The same procedure was followed for the frowning actors A and B. Participants had to indicate for each morphed identity whether it resembled more closely agent A (who had a positive disposition toward the observer) or agent B (who had a negative disposition toward the observer). The rationale was that when participants had implicitly learned that identity A had a positive, and identity B a negative, disposition toward them, then they would be more likely to judge the smiling morph (containing 50% of A and 50% of B, both smiling maximally) as more similar to identity A, and the frowning morph (containing 50% of A and 50% of B, both frowning maximally) as more similar to identity B. This is expected, as, intuitively, they would associate identity A with a positive, and identity B with a negative, valence.

Whether or not the participant had consciously detected the cue-identity contingencies was determined in a short debrief session, in which a series of questions were asked probing any awareness of the contingencies. Participants who had detected the contingencies were removed from the analysis.

### 3.2 Non-social version

#### 3.2.1 Acquisition phase

Two different shapes, a square and a circle, were used as the equivalent of the two identities A and B. The color of these objects – light blue or dark blue – was used as the equivalent of facial expressions of joy and anger, respectively. Therefore, a smooth dynamic change from light-blue to dark-blue, and vice versa, served as the equivalent of the changes in facial expression between joy and anger. A small red object positioned at the top or bottom position inside the bigger blue object, served as the equivalent of gaze direction (toward and away, respectively), while the dynamic, smooth up-or down-ward movement of this red object within the larger blue object served as the equivalent of the change in eye gaze direction. Vertical, rather than horizontal, movements were chosen to avoid interpretation of the small red objects as eyes, which might bestow the stimulus with animacy. Half of the clips started with the light-blue square, with the small red object at the top, which then gradually morphed into a dark-blue square with the small red object at the bottom (also played backwards). The other half of the clips started with the light-blue circle with the small red object at the bottom, which gradually moved into a dark-blue circle with the small object at the top (also played backwards).

#### 3.2.2 Test phase

The nonsocial test phase was, as far as possible, equivalent to its social counterpart: morphs of the square and circle were presented (in 5% steps), in either a dark-blue (≈ anger) or light-blue (≈ happy) color, with the small red object shown at the top, or bottom, of this morphed object (producing four different morph configurations; see Figure 1, Test phase nonsocial). Target objects were flanked by the two original objects, which were shown in a color that was exactly midway the light-and dark-blue colors of the Acquisition phase (≈ neutral expression, midway happy and angry). Participants had to indicate whether the target object resembled more closely object A or object B. The rationale was that when participants had implicitly learned the specific contingencies, then they would judge the morphed target object to be more similar to a circle if the little red object was *(i)* at the top of the dark-blue target object, or *(ii)* at the bottom of the light-blue target object. Similarly, they would judge the target as more similar to a square if the small red object was at the top of the light-blue target, or at the bottom of the dark-blue target. As in the social condition, a debrief was held in which questions probed awareness of the stimulus contingencies. This showed that, similar to the social version, none of the participants had detected the stimulus contingencies.

In principle, this paradigm is open to a simpler type of learning, namely *perceptual* learning. That is, it could be that participants explicitly remembered the perceptual image of for example agent A with a smile and forward directed gaze. When, in the test phase, the morphed target is shown with a smile and eyes directed forward, then this could trigger the perceptual image of agent A with a smile looking forward. This would mean that the participant could give the correct response (‘agent A’) without having implicitly learned that agent A holds a positive disposition toward the observer. To avoid this possibility, the morphed target of the test phase was shown with the eyes covered by dark sunglasses obscuring visibility of eye gaze direction (Figure 1; note that the dark sunglasses did not prevent the morphed agent from expressing a disposition).

### 3.3 Preliminary results and hypotheses for future studies

**Figure 2:**
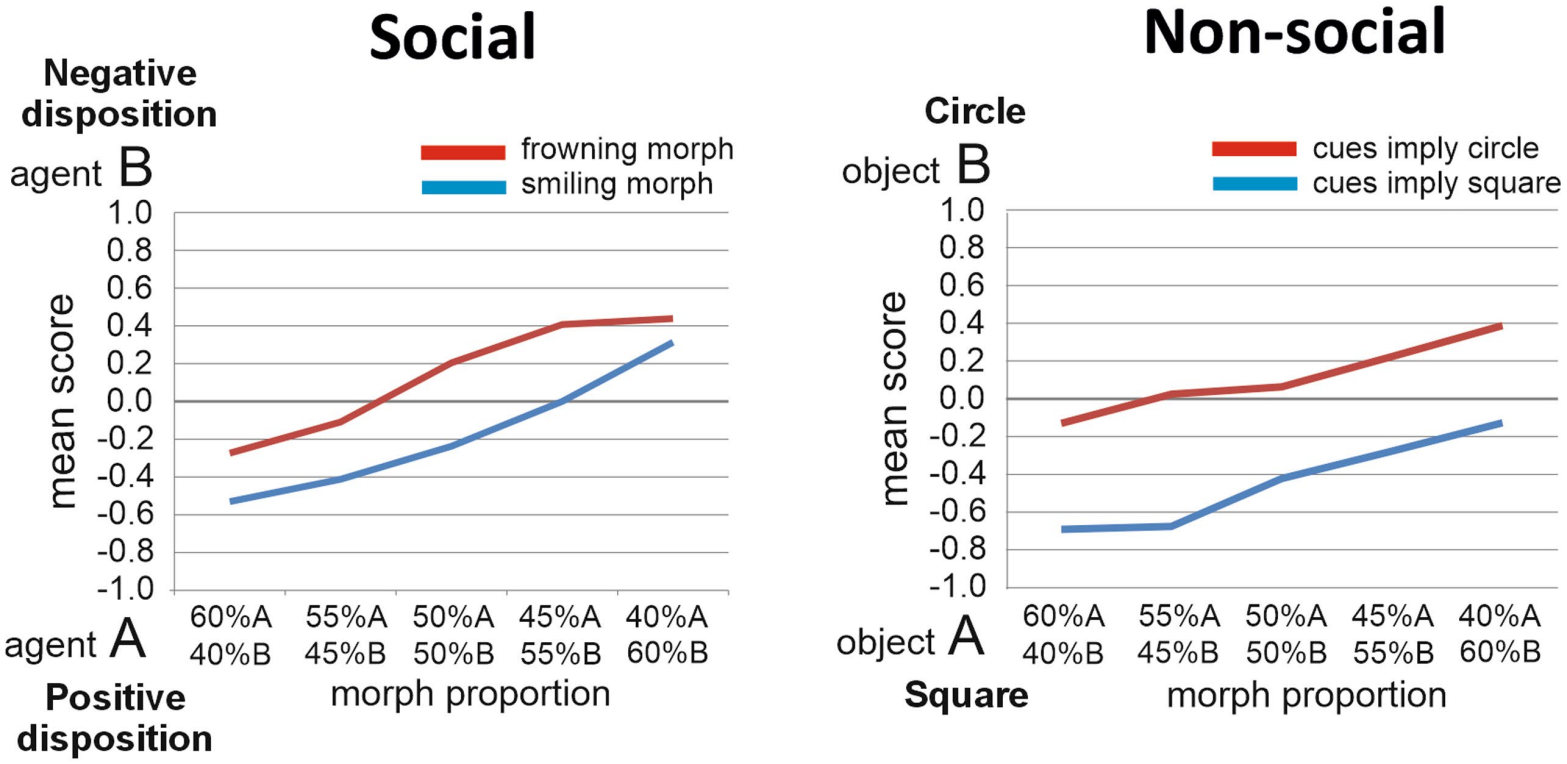
Task performance. The judgments made by TD individuals regarding the morphed target stimuli are presented for the Social implicit learning task (left panel) and for the Non-social implicit learning task (right panel).

We present here unpublished results based on 61 TD individuals who performed the Social task and on 65 participants who performed the Nonsocial task (37 of them performed both tasks; Social task: Age, M = 21.5, SD = 5.6; 25 males; Nonsocial task: Age, M = 20.7, SD = 4.7; 22 males; Figure 2). Two-way repeated measures ANOVAs were performed on the participants’ judgments in both tasks. In the Social task, the two factors were Disposition (two levels: negative disposition or anti-social vs. positive disposition or pro-social) and Proportion (five levels: proportions of agents A and B contained in the Target face, ranging from 60%A/40%B, to 40%A/60%B, in steps of 5%). The main effect of Disposition was significant [*F*(1, 60) = 8.2, *p* = 0.006, *η_p_^2^* = 0.12], reflecting that smiling Target faces were judged to resemble more the agent with the pro-social than the agent with the anti-social disposition, while frowning target faces were judged to resemble more the agent with anti-social than with pro-social disposition. The main effect of Proportion was also significant [*F*(4, 240) = 32.7, *p* < 0.001, *η_p_^2^* = 0.35]. The Disposition by Proportion interaction effect was non-significant [*F*(4, 240) = 0.99, *p* = 0.42, *η_p_^2^* = 0.016]. In the Non-social task, the two factors were Object (two levels: square vs. circle) and Proportion (five levels: proportions of objects A and B contained in the Target object, changing in steps of 5%). A similar pattern emerged as for the Social task, with significant main effects for Object [*F*(1, 64) = 41.6, *p* < 0.001, *η_p_^2^* = 0.39] and Proportion [*F*(4, 256) = 15.9, *p* < 0.001, *η_p_^2^* = 0.20], and no significant interaction effect [*F*(4, 256) = 0.83, *p* = 0.51, *η_p_^2^* = 0.013]. Thus, in both tasks, significant implicit learning effects were found.

Since the participants’ Autism Quotient scores (AQ; Baron-Cohen et al., 2001) had also been measured, we further explored any influence of AQ scores on implicit learning ability. AQ scores indicate the extent to which someone possesses autistic-like traits (ranging from 1 to maximally 50; higher scores reflecting a higher extent of autistic-like traits). Previous work indicated that the TD samples scoring low and high on the AQ may perform significantly different on social cognition tasks (Burnett and Jellema, 2013; Macinska and Jellema, 2022). The 61 TD individuals who performed the Social task had a mean AQ score of 17.6 (SD = 6.8, range 5 to 32); the 65 individuals who performed the Non-social task had a mean AQ score of 18.0 (SD = 6.5, range 7 to 32). To explore the hypothesis that autistic traits have a differential influence on implicit social/affective learning versus non-social learning, we computed the Pearson correlation coefficient to assess the linear relationship between AQ scores and a measure of implicit learning for both the Social and Nonsocial tasks. For the Social task, this measure was defined as the difference between the scores obtained for the 50%A/50%B frowning and smiling Targets. For the Nonsocial task, this measure was the difference between the 50%A/50%B light-blue and dark-blue colored Targets. Intriguingly, there was a significant negative correlation for the Social task [*r*(62) = −0.36, *p* = 0.004, two-tailed], indicating that autistic traits influenced the social/affective implicit learning ability (individuals having more traits being worse at it), while there was no significant correlation for the Nonsocial task [*r*(62) = −0.052, *p* = 0.69, two-tailed], indicating that autistic traits did not influence the nonsocial implicit learning ability. Future experiments will test individuals with ASC on these tasks. Our hypothesis is that the trend described above showing poorer implicit social/affective learning in the TD individuals with higher AQ scores, will become more prominent in individuals with ASC, while implicit learning in the nonsocial task will remain unaffected by ASC (Figure 2).

This outcome would suggest that a failure of the ASC group to implicitly learn in the social condition would not be due to an inability to implicitly learn cue contingencies *per se* (they are hypothesized to perform as well as the TD participants in the non-social condition), but may be related to an impairment to learn implicitly on the basis of affective valences.

A possible alternative explanation for the results, which does not assume an impairment in implicit affective learning in individuals high in autistic traits, is anomalous monitoring of the other’s eye gaze. If these participants would avoid looking at the agent’s eyes, or would avoid looking at direct gaze, then that would have repercussions for learning of the contingencies. Therefore, the scanning patterns of these facial expressions were examined in both TD and ASC groups in a separate study (Macinska et al., 2023). The study focused on eye-tracking using visual stimuli identical to those described here, but did not involve a test for implicit social learning. It revealed that both the TD and ASC groups spent most of their time fixating the agent’s eyes, irrespective of gaze direction, which renders this explanation unlikely.

Another alternative explanation might be that individuals with low and high AQ scores differed in their memory for the facial expressions for particular gaze directions (i.e., an interaction between expression and gaze). For example, if the individuals high in autistic traits were poor in remembering facial expressions of anger when the gaze was directed at them, but were fine with remembering expressions of anger with gaze directed away, then that might go some way in explaining the results. However, in a study specifically addressing memory for facial expressions and possible modulation by gaze direction, using identical visual stimuli (Macinska and Jellema, 2022), autistic participants remembered the facial expressions of previously encountered persons as well as TD participants, without any interaction effects with gaze direction. Note that implicit learning was not measured in Macinska and Jellema (2022). Thus, this explanation can also be excluded, leaving the impaired implicit social-affective learning explanation as a viable option.

It is further possible that the agent’s positive affect directed at the observer acted as a reward, which might have facilitated implicit learning. Such an effect would then be expected to be less pronounced in individuals who are somehow less susceptible to social rewards. It has been argued that individuals with ASC might possess a lowered ability to implicitly associate a reward value to a social stimulus, resulting in reduced social motivation (Dawson et al., 2005; Chevallier et al., 2012; Panasiti et al., 2015). Remarkably, these processes may be influenced by gender. In an fMRI (functional magnetic resonance imaging) study, Lawrence et al. (2020), using an instrumental implicit learning task, found reduced sensitivity to social reward (i.e., smiling faces) in frontostriatal and limbic structures in boys, but not in girls, with ASC. Reduced sensitivity to social reward in individuals high in autistic traits could in principle have contributed to our results. However, the majority of the participants in that group were female and thus, presumably, were sensitive to social reward.

Finally, it is possible that the presentation of dynamic facial expressions might have biased against implicit learning in individuals high in autistic traits, as it has been reported that the perception of dynamic, but not static, facial expressions, reduced activity in the amygdala and fusiform gyrus in ASC individuals (Pelphrey et al., 2007). However, the clips’ initial and final static frames, presented for 750 ms each, were most informative for implicit learning about the agent’s disposition, and we therefore do not expect this to be a major factor.

Which aspects of social intuition, as we defined it, does the newly designed task tap into? We described the social intuition ability as ‘the skill that allows one to make rapid, assumptions about what actions others are likely to take and what their intentions, emotions and thoughts might be’. We further argued that this is mainly achieved through implicit learning of regularities and contingencies between bodily articulations, contextual cues, and the immediate consequences (in terms of subsequent actions and/or reward/punishment)’. The ability purportedly measured by the current task supports the sub-part of social intuition concerned with unconsciously associating a positive or negative valence with a particular identity on the basis of the implicit learning of specific contingencies between bodily articulations performed by that identity. In the new paradigm, the main bodily articulation consisted of a gradual change of an angry facial expression into a happy one, or vice versa. A change into a happy expression, however, does not necessarily result in the agent having a positive disposition toward the observer. For that to happen, the change in facial expression needed to be accompanied by another bodily articulation: a simultaneous change in gaze direction toward the observer (a change in opposite direction would not have led to a positive disposition toward the observer).

## 4 Neural basis of social intuition

The neural basis of social cognition has been the subject of extensive investigation, largely focusing on the deliberate, conscious, social cognitive processes (e.g., Frith, 2007; Adolphs, 2009). However, the neural basis of specifically social intuition, which is largely automatic and unconscious in nature and comprises of processes leading to decisions, responses and insights that are predominantly based on the perception of others’ bodily articulations, has received much less attention. Hence, the current state of knowledge of the neural basis of social intuition is limited (*cf.* Wachowicz, 2020).

We posit here that the neural basis of social intuition may be linked to one of the key functions attributed to the mirror neuron mechanism (MNM; Rizzolatti and Craighero, 2004). The MNM ‘matches’ the visual description of another’s bodily articulation or action (either visually perceived or imagined) with an activation of those cortical motor circuits that are responsible for the *execution* of that same action, without it resulting in an overt execution of the action. The motor activity, as it were, mimics, or mirrors, the perceived (or imagined) action. How the ‘matching’ between observed and executed actions comes about remains to be elucidated, but associative learning processes are likely to be involved. Some even argue that the ‘mirroring’ is caused entirely by associative learning processes, starting from a very early age, since the baby/infant tends to look at their own actions, for example to guide their actions to a desired goal (Heyes, 2010). According to the latter view, the functional significance of the mirroring process may be limited, as it is seen as merely a by-product of associative learning. However, the ‘accidental’ forming of connections between matching visual and motor representations may in fact, rather than being a useless by-product, have far-reaching consequences. Furthermore, the contribution of associative learning processes does not exclude the possibility that brains are programmed to project visual descriptions of others’ actions (perceived or imagined), represented in the superior temporal sulcus (STS; Jellema and Perrett, 2003a,b; Pitcher and Ungerleider, 2021), onto the brain substrate that generates the execution of that action (without there being any intention to execute the action). The STS indeed projects heavily onto the parietal areas of the MNM (e.g., Rizzolatti and Luppino, 2001; Rozzi et al., 2006). In addition, the inferior temporal lobule projects to parietal mirror areas conveying information about the identity of the agent/object involved in the action (Borra et al., 2008).

Crucial for the proposed role of the MNM in social intuition is that the internal ‘mimicking’ of the observed action automatically makes a wealth of information available to the observer. That is, the off-line ‘execution’ of the observed action activates, apart from the motor representation of the action, also a range of action-associated areas. When one executes a particular action, numerous action-associated aspects, such as the accompanying visceral states (Critchley and Harrison, 2013), the action’s effect or outcome in terms of reward or punishment (Prinz, 1997), the somatosensory feedback, and the most likely subsequent action (Braukmann et al., 2017) and/or response by another individual (Hunnius and Bekkering, 2010), all occur close in time to the action execution, and therefore get linked to the action representation. Thus, the mere observation of an action will make these linked aspects instantly available to the observer, which helps to immediately ‘understand’ the action in terms of its direct consequences and affective significance. In this view, the ‘mirroring’ may subserve the immediate, automatic, understanding of other’s bodily articulations ‘from within’ (Rizzolatti and Sinigaglia, 2013, 2023), on the basis of one’s own motor repertoires, experiences and feelings. The term ‘understanding from within’ alludes to the experiential nature of the neural processes involved, in contrast to a deliberate, effortful, inferential understanding of others, which is usually indicated as explicit Theory of Mind. Thus, one could envisage the MNM as part of the neural substrate for social intuition, enabling the quick ‘understanding’ of others’ actions and gestures, on the basis of merely observing them.

### 4.1 Neural basis of action anticipation

Another reason for suggesting that the MNM may be part of the neural substrate for social intuition is that there are strong indications that it is sensitive to others’ upcoming actions – i.e. action anticipation – on the basis of contextual cues that signal that the action is forthcoming (Urgesi et al., 2006; Kilner et al., 2007; Southgate et al., 2009; Maranesi et al., 2014; Braukmann et al., 2017; Krol et al., 2020). As discussed above, the ability to automatically anticipate others’ actions is a crucial component of social intuition. Its neural basis has been investigated, in particular, using EEG (Electroencephalography), due to its superior temporal resolution (anticipation effects may be short-lived, < 1 s; Maranesi et al., 2014). In particular, suppression of the power of the mu rhythm (8–13 Hz), which can be recorded over sensorimotor and parietal cortex, has been taken as an index for MNM activity (thus, the larger the suppression the larger the neural activation). The main reason for this latter assumption is that Mu power suppressions occur both during the execution and observation of actions (see Fox et al. (2016) and Hobson and Bishop (2016, 2017), for critical evaluations of the supposed Mu-MNM link). Initially, it was thought that the MNM activates exclusively *during* the course of observed actions (1-to-1 resonation), but evidence is now accumulating that it also activates during the anticipation of upcoming predictable actions (Umiltà et al., 2001; Urgesi et al., 2006; Kilner et al., 2007; Csibra, 2008; Southgate et al., 2009; Maranesi et al., 2014; Krol et al., 2020). Some studies reported anticipatory power suppressions in the Beta frequency range (13–25 Hz; e.g. Braukmann et al., 2017; Monroy et al., 2019), rather than in the alpha range. In Braukmann et al. (2017), participants were presented with transient actions (e.g., making a cup of tea) consisting of three distinct action steps increasing in predictability: whereas the goal of step one was ambiguous, the goal of step three was highly predictable. The main finding was that predictive motor system activation, as indexed by beta-band attenuation, increased with increased predictability, with strongest activation prior to the final, most predictable, step. Intriguingly, there are indications that the Mu anticipation effects are much stronger in response to real-world actions presented by a live actor (Krol et al., 2020), as compared to actions presented in videos (Krol and Jellema, 2022, 2023). It is yet unclear why that is the case, but possibly the observer’s engagement with the observed action plays a role.

It remains to be explored whether individuals with impaired social intuition, such as those with ASC, show less anticipatory mu suppression. If so, then that would be another indication for the involvement of the MNM in social intuition. With respect to Mu suppression *during* the observation of actions there are conflicting findings, There are reports of significantly weakened Mu suppression in ASC (e.g., Bernier et al., 2007; Oberman et al., 2013; Dumas et al., 2014), but others reported no difference (e.g., Fan et al., 2010; Ward et al., 2021). Overall there seems to be a trend toward weaker Mu suppression in individuals with ASC compared to TD individuals. The variation can be due to various factors, such as participant age, heterogeneity of the ASC sample, small number of participants, use of intransient actions or static images, or the use of a separate, rather than a within-trial, baseline.

Action anticipation is intrinsically linked to the action-chain organization of motor cortex (Fogassi et al., 2005). Goal-directed actions typically can be delineated as a sequence of discrete sub-actions, and each of these sequences is engrained in motor cortex in a unique manner (Fogassi et al., 2005). This means that neurons coding for say grasping an object to bring it to the mouth are different ones from neurons encoding an identical grasping action, but forming part of another action-chain, such as grasping the object to place it away. This seems an uneconomical way to represent action-chains in motor cortex, but it means that the entire action-chain can be triggered almost instantaneously, allowing the observer a peek into the future and a sense of the action’s goal. Thus, the observer does not need to perform any deliberate, effortful thinking to figure out the next stage of the action, but just ‘sees’ it, as is typical for social intuition. Disruption of the automatic unfolding of action-chains might underpin impairments in reading the goal of others’ actions. Cattaneo et al. (2007) showed, using electromyography recordings, that in children with ASC the automatic unfolding of action-chains indeed seems to be compromised.

Though EEG is well able to capture the relatively fast (< 1 s) anticipation effects, it lacks the spatial resolution to delineate the sensorimotor, parietal and occipital sources of the power suppressions in the 8-13 Hz frequency band. Therefore, fNIRS (functional near-infrared spectroscopy; Chiarelli et al., 2017), which depends on the relatively “sluggish” BOLD (blood oxygen level dependent) response, but offers good spatial resolution, in combination with EEG would be a way forward. Like EEG, but unlike fMRI, fNIRS allows real-world live action presentations, which seem a prerequisite to evoke Mu anticipation effects.

Recent studies suggest that anticipatory MNM activity can be triggered not only by explicitly learned information (such as a specific color signal, indicating that an action is upcoming, Krol et al., 2020), but, crucially, also by implicitly learned information (e.g., Braukmann et al., 2017; Monroy et al., 2019). This is highly relevant as it further supports the candidacy of the MNM as neural substrate for social intuition, since, as we argued, implicitly learned information forms the backbone of social intuition. In Monroy et al. (2019), in the acquisition phase, infants were shown videos displaying a hand performing six different actions uniquely directed at six different toys. Trials consisted either of deterministic action pairs (e.g., action A was always followed, after a 1 s delay, by action B) or of random pairs (e.g., action C was followed, after the 1 s delay, by any of the other five actions). In the test phase, the authors found that motor activity during the 1 s delay interval selectively increased in anticipation of deterministic actions and not prior to random actions. Given their mean age of 18 months, their learning might be classed as implicit learning.

We see the MNM as an important hub in the neural network supporting social intuition. This network likely includes various social brain areas (Frith, 2007; Adolphs, 2009; Yang et al., 2015), but, crucially, also areas involved in implicit learning, possibly including the caudate and putamen in the basal ganglia (Lieberman, 2000), and areas representing reward and feelings, such as the ventral striatum (e.g., Sims et al., 2014), amygdala (e.g., Stanley et al., 2008) and anterior insular cortex (e.g., Lamm and Singer, 2010).

## 5 Concluding remarks

The traditional non-social implicit learning paradigms, as implemented in for example the SRT and AGL tasks, do not allow to draw conclusions about the role of implicit learning processes in social intuition, as they lack a number of vital characteristics associated with social intuition, such as bodily articulations, affective valences and attitudes/dispositions. The premise put forward in this article is that social intuition is largely based on the implicit learning of associations between the other’s bodily articulations and the consequences or outcomes in terms of subsequent actions and events, and associated affective valences. This means that: (i) the underlying structures of the learning processes may be relatively simple, and may not resemble the very complex structures that underpin the traditional tasks. (ii) Learning processes should be agile and relatively rapid, and should not require many hundreds of trials as in the traditional tasks. (iii) Affective valences should be incorporated in the paradigm, as these are a defining characteristic of social intuition (Cleeremans, 2011).

Attempts to ‘socialise’ the traditional AGL task have recently been undertaken (e.g., Jurchis et al., 2023; Jurchiș and Dienes, 2023), but these paradigms are still rather non-ecologically-valid and do not incorporate affective valences. The studies by Macinska and Jellema, highlighted in this article, are another attempt to address this issue. In this paradigm positive or negative valences are unconsciously associated with a particular identity, on the basis of implicitly learned contingencies between bodily articulations performed by that identity. This approach seems able to discriminate between individuals low and high in autistic traits. Whether an impairment in implicit affective learning in ASC, as suggested by these studies, indeed has consequences for the development of their social intuition skills remains to be confirmed. It should be noted, however, that while the Macinska and Jellema paradigm is more ecologically valid than other paradigms, it is still not really reminiscent of a social *interaction*. Further, a risk associated with the latter paradigm is that other forms of learning, such as perceptual learning, could possibly interfere with the results.

The cues on which implicit social learning is based consist, to a large extent, of bodily articulations (gestures, actions, facial expressions, eye gaze direction, vocalizations), all of which are specifically represented in the MNM. A major premise of this article is that the MNM is a crucial hub in the neural social intuition network. This, however, poses an interesting problem. According to the extensive literature on the MNM (e.g., Rizzolatti and Craighero, 2004), the main input to the MNM consists of the visual description of the other’s action, which causes motor resonation in the MNM, leading to activation of associated areas that represent consequences of the observed action. These associated areas are those areas that would have been activated if the observer themselves would have carried out that action, and are therefore intricately linked to the action representation. Such consequences may consist of the most likely subsequent action/event, visceral states, affective states, sensory feedback and possible reward/punishment. This means that mere observation of the action makes these outcomes directly ‘available’ to the observer, and lead to what Rizzolatti and Sinigaglia (2013, 2023) called an ‘experiential’ understanding of the action (or understanding ‘from within’).

The interesting problem is that the ‘immediate insight’ we commonly call social intuition pertains to the other individual’s attitude/disposition, not to the observer’s own attitude/disposition; these two may differ profoundly. How then can motor resonation in the MNM bridge this gap? We propose that implicit social learning plays a crucial role. When the observation of the agent performing a particular action in a particular context does not trigger any implicitly learned information, then the observer’s immediate social intuition regarding that agent will indeed be determined by their own repertoire of experiences (i.e., by what they would feel/do if they would carry out that action in that context). If, however, implicitly learned information is available and triggered, then that information will inform and dominate the observer’s social intuition. The implicitly learned information may be linked to a particular individual, as in the paradigm presented in this article (note: identity information, originating in inferior temporal gyrus, is conveyed to the parietal MNM areas), or may be linked to individuals in general. The automatic use of information derived from implicit social learning may lead to accurate social intuition; without it an inaccurate, own-experience-centered, type of social intuition prevails. Possibly, social intuition in ASC individuals is too reliant on the latter pathway.

Implicit social learning also pertains to contextual cues that herald upcoming actions. In these cases, the cues themselves, rather than the observation of an action, trigger motor activity in the MNM. We argue that automatic action anticipation is a crucial contributor to social intuition, enabling the rapid judgments and responses made during fast-paced social interaction. The MNM provides a candidate neural substrate for such anticipatory representations of others’ actions (e.g., Kilner et al., 2007; Krol et al., 2020). Importantly, anticipatory MNM activity can be triggered by implicitly learned information (e.g., Braukmann et al., 2017; Monroy et al., 2019). These findings need, however, be backed up by more research. Whether MNM activity in anticipation of others’ actions is reduced in ASC remains to be clarified.

Finally, it should be noted that a limitation of the treatment of social intuition in this article is that it only deals with its automatic nature. For any human social intuition capacity to be meaningful in real-life social situations, higher-level knowledge and explicit, deliberate Theory-of-Mind (ToM) processes are required as well. Moreover, explicit ToM processes might interact with, or modulate, automatic processes (*cf.*, Wincenciak et al., 2022), such as social intuition. For example, one could speculate that when in our implicit social learning paradigm the participant *knows* that, even though they can see the agent, the agent cannot see them (because of one-way mirror), they might not implicitly learn to associate the agent with a positive or negative disposition toward them (even though the agent was looking right at them). MNM activation might also be sensitive to explicit ToM reasoning. For example, where observers might show anticipatory MNM activity when an agent’s gaze is directed at a cup of coffee, which implicitly signals they are likely to grasp the cup and drink, they may not show this anticipatory MNM activity if they understood that the agent believed that the cup contained some other non-desired liquid. Such possible interactions between explicit ToM and automatic social intuition remain to be explored in future work.

## Data availability statement

The datasets presented in this article are not readily available because they constitute preliminary data. Requests to access the datasets should be directed to T.Jellema@hull.ac.uk.

## Ethics statement

The studies involving humans were approved by Ethics committee of the Faculty of Health Sciences of the University of Hull. The studies were conducted in accordance with the local legislation and institutional requirements. The participants provided their written informed consent to participate in this study.

## Author contributions

TJ: Conceptualization, Writing – original draft. SM: Writing – review & editing. RO’C: Writing – review & editing. TS: Writing – review & editing.

## References

[ref1] Adolphs R. (2009). The social brain: neural basis of social knowledge. Annu. Rev. Psychol. 60, 693–716. doi: 10.1146/annurev.psych.60.110707.163514, PMID: 18771388 PMC2588649

[ref2] Aizenstein H. J. Stenger V. A. Cochran J. Clark K. Johnson M. Nebes R. D.. (2004). Regional brain activation during concurrent implicit and explicit sequence learning. Cereb. Cortex 14, 199–208. doi: 10.1093/cercor/bhg119 14704217

[ref3] Amso D. Davidow J. (2012). The development of implicit learning from infancy to adulthood: item frequencies, relations, and cognitive flexibility. Dev. Psychobiol. 54, 664–673. doi: 10.1002/dev.20587, PMID: 22714674

[ref4] Bargh J. A. (1994). “The four horsemen of automaticity: awareness, intention, efficiency, and control in social cognition” in Handbook of social cognition. eds. Wyer R. S. Srull T. K., vol. 1 (Hillsdale, NJ: Erlbaum), 1–40.

[ref5] Barnes K. A. Howard J. H. Howard D. V. Gilotty L. Kenworthy L. Gaillard W. D.. (2008). Intact implicit learning of spatial context and temporal sequences in childhood autism spectrum disorder. Neuropsychology 22, 563–570. doi: 10.1037/0894-4105.22.5.563, PMID: 18763876

[ref6] Baron-Cohen S. Wheelwright S. Skinner S. Martin J. Clubley E. (2001). The autism Spectrum quotient (AQ): evidence from Asperger syndrome/high functioning autism, males and females, scientists and mathematicians. J. Autism Dev. Disord. 31, 5–17. doi: 10.1023/A:1005653411471 11439754

[ref7] Bayliss A. P. Tipper S. P. (2006). Predictive gaze cues and personality judgments. Psychol. Sci. 17, 514–520. doi: 10.1111/j.1467-9280.2006.01737.x, PMID: 16771802 PMC2080823

[ref8] Bechara A. Damasio H. Tranel D. Damasio A. R. (1997). Deciding advantageously before knowing the advantageous strategy. Science 275, 1293–1295. doi: 10.1126/science.275.5304.1293, PMID: 9036851

[ref9] Bernier R. Dawson G. Webb S. Murias M. (2007). EEG mu rhythm and imitation impairments in individuals with autism spectrum disorder. Brain Cogn. 64, 228–237. doi: 10.1016/j.bandc.2007.03.004, PMID: 17451856 PMC2709976

[ref10] Borra E. Belmalih A. Calzavara R. Gerbella M. Murata A. Rozzi S.. (2008). Cortical connections of the macaque anterior intraparietal (AIP) area. Cereb. Cortex 18, 1094–1111. doi: 10.1093/cercor/bhm146 17720686

[ref11] Braukmann R. Bekkering H. Hidding M. Edita Poljac E. Buitelaar J. K. Hunnius S. (2017). Predictability of action sub-steps modulates motor system activation during the observation of goal-directed actions. Neuropsychologia 103, 44–53. doi: 10.1016/j.neuropsychologia.2017.07.009 28716611

[ref12] Brown J. Aczel B. Jiménez L. Kaufman S. B. Grant K. P. (2010). Intact implicit learning in autism spectrum conditions. Q. J. Exp. Psychol. 63, 1789–1812. doi: 10.1080/17470210903536910, PMID: 20204919

[ref13] Burnett H. G. Jellema T. (2013). (re-)conceptualisation in Asperger’s syndrome and typical individuals with varying degrees of autistic-like traits. J. Autism Dev. Disord. 43, 211–223. doi: 10.1007/s10803-012-1567-z 22743806

[ref14] Cattaneo L. Fabbri-Destro M. Boria S. Pieraccini C. Monti A. Cossu G.. (2007). Impairment of actions chains in autism and its possible role in intention understanding. Proc. Natl. Acad. Sci. U S A 104, 17825–17830. doi: 10.1073/pnas.0706273104, PMID: 17965234 PMC2077067

[ref15] Chevallier C. Kohls G. Troiani V. Brodkin E. S. Schultz R. T. (2012). The social motivation theory of autism. Trends Cogn. Sci. 16, 231–239. doi: 10.1016/j.tics.2012.02.007 22425667 PMC3329932

[ref16] Chiarelli A. M. Zappasodi F. Di Pompeo F. Merla A. (2017). Simultaneous functional near-infrared spectroscopy and electroencephalography for monitoring of human brain activity and oxygenation: a review. Neurophotonics 4:041411. doi: 10.1117/1.NPh.4.4.041411, PMID: 28840162 PMC5566595

[ref17] Cleeremans A. (2011). The radical plasticity thesis: how the brain learns to be conscious. Front. Psychol. 2:86. doi: 10.3389/fpsyg.2011.00086 21687455 PMC3110382

[ref18] Costea A. R. Jurchiș R. Visu-Petra L. Cleeremans A. (2023). Implicit and explicit learning of socio-emotional information in a dynamic interaction with a virtual avatar. Psychol. Res. 87, 1057–1074. doi: 10.1007/s00426-022-01709-4 36036291 PMC10191928

[ref19] Critchley H. D. Harrison N. E. (2013). Visceral influences on brain and behavior. Neuron 77, 624–638. doi: 10.1016/j.neuron.2013.02.008 23439117

[ref20] Csibra G. (2008). Action mirroring and action understanding: an alternative account. Sensorymotor foundations of higher cognition. Attention Performance XXII, 435–459. doi: 10.1093/acprof:oso/9780199231447.003.0020

[ref21] Damasio A. R. (1994). Descartes’ error: Emotion, reason, and the human brain. New York: G.P. Putnam’s Sons.

[ref22] Dawson G. Webb S. J. McPartland J. (2005). Understanding the nature of face processing impairment in autism: insights from behavioral and electrophysiological studies. Dev. Neuropsychol. 27, 403–424. doi: 10.1207/s15326942dn2703_6 15843104

[ref23] De Houwer J. Moors A. (2012). “How to define and examine implicit processes?” in Implicit and explicit processes in the psychology of science. eds. Proctor R. Capaldi J. (New York: OUP USA), 183–198.

[ref24] Dienes Z. Scott R. (2005). Measuring unconscious knowledge: distinguishing structural knowledge and judgment knowledge. Psychol. Res. 69, 338–351. doi: 10.1007/s00426-004-0208-3 15944859

[ref25] DSM-V-TR (2022). Diagnostic and statistical manual of mental disorders (5th ed., text revision). Washington, DC: American Psychiatric Publishing.

[ref26] Dumas G. Soussignan R. Hugueville L. Martinerie J. Nadel J. (2014). Revisiting mu suppression in autism spectrum disorder. Brain Res. 1585, 108–119. doi: 10.1016/j.brainres.2014.08.035, PMID: 25148709

[ref27] Evans J. B. T. (2003). In two minds. Trends Cogn. Sci. 7, 454–459. doi: 10.1016/j.tics.2003.08.012, PMID: 14550493

[ref28] Fan Y. T. Decety J. Yang C. Y. Liu J. L. Cheng Y. (2010). Unbroken mirror neurons in autism spectrum disorders: Unbroken mirror neurons in ASD. J. Child Psychol. Psychiatry 51, 981–988. doi: 10.1111/j.1469-7610.2010.02269.x 20524939

[ref29] Fogassi L. Ferrari P. F. Gesierich B. Rozzi S. Chersi F. Rizzolatti G. (2005). Parietal lobe: from action organization to intention understanding. Science 308, 662–667. doi: 10.1126/science.1106138 15860620

[ref30] Foti F. de Crescenzo F. Vivanti G. Menghini D. Vicari S. (2015). Implicit learning in individuals with autism spectrum disorders: a meta-analysis. Psychol. Med. 45, 897–910. doi: 10.1017/S0033291714001950, PMID: 25126858

[ref31] Fox N. A. Bakermans-Kranenburg M. J. Yoo K. H. Bowman L. C. Cannon E. N. Vanderwert R. E.. (2016). Assessing human mirror activity with EEG mu rhythm. Psychol. Bull. 142, 291–313. doi: 10.1037/bul0000031, PMID: 26689088 PMC5110123

[ref32] Frith C. D. (2007). The social brain? Philos. Trans. R. Soc. B 362, 671–678. doi: 10.1098/rstb.2006.2003, PMID: 17255010 PMC1919402

[ref33] Frith C. D. (2008). Social cognition. Philos. Trans. R. Soc. B 363, 2033–2039. doi: 10.1098/rstb.2008.0005, PMID: 18292063 PMC2375957

[ref34] Gómez J.-C. Kersken V. A. Ball D. N. Seed A. M. (2017). Knowing without knowing. Stud. Psychol. 38, 37–62. doi: 10.1080/02109395.2016.1268389

[ref35] Gordon B. Stark S. (2007). Procedural learning of a visual sequence in individuals with autism. Focus Autism Other Dev. Disabil. 22, 14–22. doi: 10.1177/10883576070220010201

[ref36] Greenwald A. Banaji M. R. (1995). Implicit social cognition: attitudes, self-esteem, and stereotypes. Psychol. Rev. 102, 4–27. doi: 10.1037/0033-295X.102.1.4, PMID: 7878162

[ref37] Hertwig R. Hoffrage U. The ABC Research group. Simple heuristics in a social world (2013). Oxford: OUP

[ref38] Heyes C. (2010). Where do mirror neurons come from? Neurosci. Biobehav. Rev. 34, 575–583. doi: 10.1016/j.neubiorev.2009.11.007 19914284

[ref39] Hobson H. M. Bishop D. V. M. (2016). Mu suppression – a good measure of the human mirror neuron system? Cortex 82, 290–310. doi: 10.1016/j.cortex.2016.03.019 27180217 PMC4981432

[ref40] Hobson H. M. Bishop D. V. M. (2017). The interpretation of mu suppression as an index of mirror neuron activity: past, present and future. R. Soc. Open Sci. 4:160662. doi: 10.1098/rsos.160662 28405354 PMC5383811

[ref41] Hudson M. Burnett H. G. Jellema T. (2012a). Anticipation of action intentions in autism Spectrum disorder. J. Autism Dev. Disord. 42, 1684–1693. doi: 10.1007/s10803-011-1410-y 22113746

[ref42] Hudson M. Liu C. H. Jellema T. (2009). Anticipating intentional actions: the effect of eye gaze direction on the judgment of head rotation. Cognition 112, 423–434. doi: 10.1016/j.cognition.2009.06.011 19615675

[ref43] Hudson M. Nijboer T. C. W. Jellema T. (2012b). Implicit social learning in relation to autistic-like traits. J. Autism Dev. Disord. 42, 2534–2545. doi: 10.1007/s10803-012-1510-3 22447071

[ref44] Hunnius S. Bekkering H. (2010). The early development of object knowledge: a study of infants’ visual anticipations during action observation. Dev. Psychol. 46, 446–454. doi: 10.1037/a0016543, PMID: 20210504

[ref45] Jellema T. Baker C. I. Wicker B. Perrett D. I. (2000). Neural representation for the perception of the intentionality of actions. Brain Cogn. 44, 280–302. doi: 10.1006/brcg.2000.1231, PMID: 11041992

[ref46] Jellema T. Lorteije J. A. M. van Rijn S. van t' Wout M. de Haan E. van Engeland H.. (2009). Involuntary interpretation of social cues is compromised in autism spectrum disorders. Autism Res. 2, 192–204. doi: 10.1002/aur.83, PMID: 19642087

[ref47] Jellema T. Pecchinenda A. Palumbo L. Tan E. G. (2011). Biases in the perception and affective valence of neutral facial expressions induced by the immediate perceptual history. Vis. Cogn. 19, 616–634. doi: 10.1080/13506285.2011.569775

[ref48] Jellema T. Perrett D. I. (2003a). Cells in monkey STS responsive to articulated body motions and consequent static posture: a case of implied motion? Neuropsychologia 41, 1728–1737. doi: 10.1016/S0028-3932(03)00175-1 14527537

[ref49] Jellema T. Perrett D. I. (2003b). Perceptual history influences neural responses to face and body postures. J. Cogn. Neurosci. 15, 961–971. doi: 10.1162/089892903770007353 14614807

[ref50] Jurchis R. Costea A. Opre A. (2023). A implicit and explicit learning of socio-emotional information are not related to the level of depressive symptomatology. Motiv. Emot. 47, 795–809. doi: 10.1007/s11031-023-10022-5

[ref51] Jurchiș R. Dienes Z. (2023). Implicit learning of regularities followed by realistic body movements in virtual reality. Psychon. Bull. Rev. 30, 269–279. doi: 10.3758/s13423-022-02175-0 36085234

[ref52] Kahneman D. (2011). Thinking, Fast and Slow. New York: Farrar, Straus and Giroux.

[ref53] Kilner J. M. Friston K. J. Frith C. D. (2007). Predictive coding: an account of the mirror neuron system. Cogn. Process. 8, 159–166. doi: 10.1007/s10339-007-0170-2 17429704 PMC2649419

[ref54] Kliemann D. Rosenblau G. Bölte S. Heekeren H. R. Dziobek I. (2013). Face puzzle – two new video-based tasks for measuring explicit and implicit aspects of facial emotion recognition. Front. Psychol. 4:376. doi: 10.3389/fpsyg.2013.00376 23805122 PMC3693509

[ref55] Krol M. A. Jellema T. (2022). Sensorimotor anticipation of others’ actions in real-world and video settings: modulation by level of engagement? Soc. Neurosci. 17, 293–304. doi: 10.1080/17470919.2022.2083229, PMID: 35613478

[ref56] Krol M. A. Jellema T. (2023). Sensorimotor representation of observed dyadic actions with varying agent involvement: an EEG mu study. Cogn. Neurosci. 14, 25–35. doi: 10.1080/17588928.2022.2084605 35699606

[ref57] Krol M. A. Schutter D. J. L. G. Jellema T. (2020). Sensorimotor cortex activation during anticipation of upcoming predictable but not unpredictable actions. Soc. Neurosci. 15, 214–226. doi: 10.1080/17470919.2019.1674688 31587597

[ref58] Kunst-Wilson W. R. Zajonc R. B. (1980). Affective discrimination of stimuli that cannot be recognized. Science 207, 557–558. doi: 10.1126/science.7352271, PMID: 7352271

[ref59] Lamm C. Singer T. (2010). The role of anterior insular cortex in social emotions. Brain Struct. Funct. 214, 579–591. doi: 10.1007/s00429-010-0251-3 20428887

[ref60] Lawrence K. E. Hernandez L. M. Eilbott J. Jack A. Aylward E. Gaab N.. (2020). Neural responsivity to social rewards in autistic female youth. Psychiatry 10. doi: 10.1038/s41398-020-0824-8, PMID: 32488083 PMC7266816

[ref61] Lieberman M. D. (2000). Intuition: A social cognitive neuroscience approach. Psychol. Bull. 126, 109–137. doi: 10.1037/0033-2909.126.1.109, PMID: 10668352

[ref62] Macinska S. M. (2019). Implicit social cognition in autism spectrum disorder. PhD Thesis. Hull: University of Hull, UK.

[ref63] Macinska S. M. Jellema T. (2016). Implicit learning of social and non-social information. Conference paper presented at fifth implicit learning seminar, Lancaster, England.

[ref64] Macinska S. M. Jellema T. (2022). Memory for facial expressions on the autism Spectrum: the influence of gaze direction and type of expression. Autism Res. 15, 870–880. doi: 10.1002/aur.2682 35150078

[ref65] Macinska S. M. Krol M. Jellema T. (2015a). Variations in implicit social learning in the typically-developed population. Perception 44:45.

[ref66] Macinska S. M. Krol M Jellema T (2015b). The capacity for implicit social learning in relation to autistic traits. Conference presentation: EuroAsianPacific joint conference on cognitive science, Turin, Italy.

[ref67] Macinska S. M. Lindsay S. Jellema T. (2023). Visual attention to dynamic emotional faces in adults on the autism spectrum. J. Autism Dev. Disord. doi: 10.1007/s10803-023-05979-8 PMC11143001 37079180

[ref68] Maranesi M. Livi A. Fogassi L. Rizzolatti G. Bonini L. (2014). Mirror neuron activation prior to action observation in a predictable context. J. Neurosci. 34, 14827–14832. doi: 10.1523/JNEUROSCI.2705-14.2014 25378150 PMC6608372

[ref69] Monroy C. D. Gerson S. A. Domínguez-Martínez E. Kaduk K. Hunnius S. Reid V. (2019). Sensitivity to structure in action sequences: an infant event-related potential study. Neuropsychologia 126, 92–101. doi: 10.1016/j.neuropsychologia.2017.05.007 28487250

[ref70] Moors A. De Houwer J. (2006). Automaticity: a conceptual and theoretical analysis. Psychol. Bull. 132, 297–326. doi: 10.1037/0033-2909.132.2.297 16536645

[ref71] Nemeth D. Janacsek K. Balogh V. Londe Z. Mingesz R. Fazekas M.. (2010). Learning in autism: implicitly superb. PLoS One 5:e11731. doi: 10.1371/journal.pone.0011731, PMID: 20661300 PMC2908691

[ref72] Nissen M. J. Bullemer P. (1987). Attentional requirements of learning. Cogn. Psychol. 19, 1–32. doi: 10.1016/0010-0285(87)90002-8

[ref73] Norman E. Price M. C. (2012). Social intuition as a form of implicit learning: sequences of body movements are learned less explicitly than letter sequences. Adv. Cogn. Psychol. 8, 121–131. doi: 10.5709/acp-0109-x, PMID: 22679467 PMC3367869

[ref74] Oberman L. M. McCleery J. P. Hubbard E. M. Bernier R. Wiersema J. R. Raymaekers R.. (2013). Developmental changes in mu suppression to observed and executed actions in autism spectrum disorders. Soc. Cogn. Affect. Neurosci. 8, 300–304. doi: 10.1093/scan/nsr097, PMID: 22302843 PMC3594721

[ref75] Palumbo L. Burnett H. G. Jellema T. (2015). Atypical emotional anticipation in high-functioning autism. Mol. Autism. 6:47. doi: 10.1186/s13229-015-0039-7, PMID: 26279832 PMC4537555

[ref76] Palumbo L. Jellema T. (2013). Beyond face value: does involuntary emotional anticipation shape the perception of facial expressions? PLoS One 8:e56003. doi: 10.1371/journal.pone.0056003, PMID: 23409112 PMC3569428

[ref77] Panasiti M. S. Puzzo I. Chakrabarti B. (2015). Autistic traits moderate the impact of reward learning on social behaviour. Autism Res. 9, 471–479. doi: 10.1002/aur.1523 26280134 PMC4949660

[ref78] Pelphrey K. A. Morris J. P. McCarthy G. Labar K. S. (2007). Perception of dynamic changes in facial affect and identity in autism. Soc. Cogn. Affect. Neurosci. 2, 140–149. doi: 10.1093/scan/nsm010, PMID: 18174910 PMC2174259

[ref79] Pitcher D. Ungerleider L. (2021). Evidence for a third visual pathway specialized for social perception. Trends Cogn. Sci. 25, 100–110. doi: 10.1016/j.tics.2020.11.006 33334693 PMC7811363

[ref80] Prinz W. (1997). Perception and action planning. Eur. J. Cogn. Psychol. 9, 129–154. doi: 10.1080/713752551

[ref81] Rauch S. L. Whalen P. J. Savage C. R. Curran T. Kendrick A. Brown H. D.. (1997). Striatal recruitment during an implicit sequence learning task as measured by functional magnetic resonance imaging. Hum. Brain Mapp. 5, 124–132. doi: 10.1002/(SICI)1097-0193(1997)5:2<124::AID-HBM6>3.0.CO;2-5, PMID: 10096417

[ref82] Reber A. S. (1967). Implicit learning of artificial grammars. J. Verbal Learn. Verbal Behav. 6, 855–863. doi: 10.1016/S0022-5371(67)80149-X

[ref83] Reber A. S. (1989). Implicit learning and tacit knowledge. J. Exp. Psychol. Gen. 118, 219–235. doi: 10.1037/0096-3445.118.3.219

[ref84] Redcay E. Schilbach L. (2019). Using second-person neuroscience to elucidate the mechanisms of social interaction. Nat. Rev. Neurosci. 20, 495–505. doi: 10.1038/s41583-019-0179-4, PMID: 31138910 PMC6997943

[ref85] Rizzolatti G. Craighero L. (2004). The mirror-neuron system. Annual review. Neuroscience 27, 169–192. doi: 10.1146/annurev.neuro.27.070203.144230 15217330

[ref86] Rizzolatti G. Luppino G. (2001). The cortical motor system. Neuron 31, 889–901. doi: 10.1016/S0896-6273(01)00423-8 11580891

[ref87] Rizzolatti G. Sinigaglia C. (2013). “Understanding action from the inside” in Action science: Foundations of an emerging discipline. eds. Prinz W. Beisert M. Herwig A. (Cambridge, MA: MIT Press)

[ref88] Rizzolatti G. Sinigaglia C. (2023). Mirroring brains: How we understand others from the inside. Oxford: Oxford University Press.

[ref89] Rozzi S. Calzavara R. Belmalih A. Borra E. Gregoriou G. G. Matelli M.. (2006). Cortical connections of the inferior parietal cortical convexity of the macaque monkey. Cereb. Cortex 16, 1389–1417. doi: 10.1093/cercor/bhj076, PMID: 16306322

[ref90] Sato W. Krumhuber E. G. Jellema T. Williams J. H. G. (2019). Editorial: dynamic emotional communication. Front. Psychol. 10:2836. doi: 10.3389/fpsyg.2019.02836, PMID: 31956318 PMC6951420

[ref91] Schilbach L. Eickhoff S. B. Cieslik E. C. Kuzmanovic B. Vogeley K. (2012). Shall we do this together? Social gaze influences action control in a comparison group, but not in individuals with high-functioning autism. Autism 16, 151–162. doi: 10.1177/1362361311409258, PMID: 21810910

[ref92] Schilbach L. Timmermans B. Reddy V. Costall A. Bente G. Schlicht T.. (2013). Toward a second-person neuroscience. Behav. Brain Sci. 36, 393–414. doi: 10.1017/S0140525X12000660, PMID: 23883742

[ref93] Senju A. Southgate V. White S. Frith U. (2009). Mindblind eyes: an absence of spontaneous theory of mind in Asperger syndrome. Science 325, 883–885. doi: 10.1126/science.1176170, PMID: 19608858

[ref94] Shiffrin R. M. Schneider W. (1977). Controlled and automatic human information processing: II. Perceptual learning, automatic attending and a general theory. Psychol. Rev. 84, 127–190. doi: 10.1037/0033-295X.84.2.127

[ref95] Sims T. B. Neufeld J. Johnstone T. Chakrabarti B. (2014). Autistic traits modulate frontostriatal connectivity during processing of rewarding faces. Soc. Cogn. Affect. Neurosci. 9, 2010–2016. doi: 10.1093/scan/nsu010 24493838 PMC4249479

[ref96] Southgate V. Johnson M. H. Osborne T. Csibra G. (2009). Predictive motor activation during action observation in human infants. Biol. Lett. 5, 769–772. doi: 10.1098/rsbl.2009.0474, PMID: 19675001 PMC2828001

[ref9001] Stanley D. Phelps E. Banaji M. (2008). The Neural Basis of Implicit Attitudes. Current Directions in Psychological Science, 17, 164–170. doi: 10.1111/j.1467-8721.2008.00568.x

[ref97] Travers B. G. Powell P. S. Mussey J. L. Klinger L. G. Crisler M. E. Klinger M. R. (2013). Spatial and identity cues differentially affect implicit contextual cueing in adolescents and adults with autism spectrum disorder. J. Autism Dev. Disord. 43, 2393–2404. doi: 10.1007/s10803-013-1787-x, PMID: 23417264

[ref98] Umiltà M. A. Kohler E. Gallese V. Fogassi L. Fadiga L. Keysers C.. (2001). I know what you are doing: a neurophysiological study. Neuron 31, 155–165. doi: 10.1016/S0896-6273(01)00337-3 11498058

[ref99] Urgesi C. Valentina M. Matteo C. Aglioti S. (2006). Mapping implied body actions in the human motor system. J. Neurosci. 26, 7942–7949. doi: 10.1523/JNEUROSCI.1289-06.2006 16870739 PMC6674209

[ref100] Wachowicz B. (2020). “The neural basis of intuitive decision making” in Advances in science, technology, higher education and Society in the Conceptual age: STHESCA. ed. Marek T. (San Diego, CA: AHFE International, USA)

[ref101] Ward E. K. Braukmann R. Weiland R. F. Bekkering H. Buitelaar J. K. Hunnius S. (2021). Action predictability is reflected in beta power attenuation and predictive eye movements in adolescents with and without autism. Neuropsychologia 157:107859. doi: 10.1016/j.neuropsychologia.2021.107859 33887295

[ref102] Wincenciak J. Palumbo L. Epihova G. Barraclough N. E. Jellema T. (2022). Are adaptation aftereffects for facial emotional expressions affected by prior knowledge about the emotion? Cognit. Emot. 36, 602–615. doi: 10.1080/02699931.2022.2031907 35094648

[ref103] Yang D. Y. J. Rosenblau G. Keifer C. Pelphrey K. A. (2015). An integrative neural model of social perception, action observation, and theory of mind. Neurosci. Biobehav. Rev. 51, 263–275. doi: 10.1016/j.neubiorev.2015.01.020 25660957 PMC4940188

[ref104] Zhang Q. Li L. Guo X. Zheng L. Wu Y. Zhou C. (2020). Implicit learning of symmetry of human movement and gray matter density: evidence against pure domain general and pure domain specific theories of implicit learning. Int. J. Psychophysiol. 147, 60–71. doi: 10.1016/j.ijpsycho.2019.10.008, PMID: 31734444

